# Associations Between Emotional Distress and Injury Occurrence in Physically Active Students

**DOI:** 10.3390/jcm15051822

**Published:** 2026-02-27

**Authors:** Jarosław Domaradzki

**Affiliations:** Department of Biological Principles of Physical Activity, Wroclaw University of Health and Sport Sciences, 51-612 Wroclaw, Poland; jaroslaw.domaradzki@awf.wroc.pl

**Keywords:** negative emotional states, depression, anxiety, stress, injury occurrence, young adults, physical activity

## Abstract

**Background/Objectives**: Negative emotional states such as depression, anxiety, and stress have been proposed as psychological correlates of injury occurrence, yet evidence regarding their independent and combined effects remains inconsistent, particularly with respect to sex differences. The present investigation focused on whether the relationship between adverse emotional states and injury occurrence differs between men and women among physically active young adults. **Methods**: The study was conducted with a cross-sectional design and included 418 university students (199 men and 219 women; mean age: men 20.73 ± 0.85 years; women 20.56 ± 0.74 years). Participants’ anthropometric characteristics included body height (men, 182.19 ± 7.10 cm; women, 168.17 ± 6.01 cm) and body weight (men, 79.63 ± 9.87 kg; women, 60.86 ± 9.05 kg). Symptoms of depression, anxiety, and stress were measured using the Depression Anxiety Stress Scale (DASS-21), and injury history within the previous 12 months was obtained via a structured self-report injury questionnaire. Logistic regression models were used to evaluate associations between emotional states and injury occurrence, including assessment of linear, non-linear, and interaction effects. Analyses were stratified by sex and adjusted for training weekly load and training experience. Complementary profile analysis was conducted to assess emotional state configurations by injury occurrence. **Results**: Linear models provided the most parsimonious representation of the associations between emotional states and injury occurrence, with no support for non-linear or interaction effects. In sex-stratified multivariable models, anxiety was modestly associated with injury occurrence in males (OR = 1.05; 95% CI: 1.00–1.11), whereas depression and stress were not significant correlates. No significant associations were observed in females. Profile analysis revealed distinct emotional dimensions but showed no differences in overall profile level or shape between injured and non-injured participants. **Conclusions**: Negative emotional states demonstrated limited and predominantly additive associations with injury occurrence. Anxiety showed a small, sex-specific association in males, while overall emotional state measures exhibited limited explanatory value for injury occurrence.

## 1. Introduction

Injuries constitute a relevant health and functional concern in young adults engaged in physical activity, including university students participating in organized sport, recreational exercise, and mandatory physical education. Sumilo et al. (2006) [1] found that nearly one in five students (18%) reported sustaining at least one injury requiring medical care within a year. Participation in team sports was associated with a 2.5-fold higher likelihood of injury. Cai et al., 2019 [2], confirmed this pattern, reporting an overall injury incidence density of 0.57 per 1000 h of exposure, with males experiencing higher rates (1.07) than females (0.45). Although injury research has traditionally focused on biomechanical load, exposure, and environmental hazards, accumulating evidence indicates that psychological factors contribute to injury susceptibility across both athletic and occupational populations. Stress–injury frameworks propose that life stress, personality traits, and coping resources shape injury occurrence through their influence on psychophysiological strain and behavioral regulation under load [3].

Evidence from prospective sport research indicates that athletes experiencing elevated mood disturbance and perceived stress are more likely to sustain injuries and present distinct injury profiles, underscoring the importance of incorporating psychological variables into injury frameworks [4,5]. Field studies in occupational settings further support a biopsychosocial perspective, demonstrating that psychological strain (e.g., job stress, PTSD, burnout) interacts with physical workload to elevate musculoskeletal symptom risk [6,7]. Study on hard-working workers showed that the highest and significant increase in musculoskeletal symptoms occurred in groups simultaneously exposed to high physical and high psychosocial workplace exposures [8]. Proposed mechanisms involving attention or motor control remain largely theoretical in this literature.

Contemporary injury models conceptualize injury causation as multifactorial, distinguishing extrinsic factors from intrinsic individual characteristics, including psychological variables measured at baseline [9].

Empirical research consistently identifies trait anxiety, stress susceptibility, coping style, and recent life stress as intrinsic psychological predictors of injury occurrence in sport and youth populations [10,11]. Psychological predictors typically explain modest proportions of injury variance and are therefore interpreted as intrinsic components within broader multifactorial models rather than standalone causal factors [12,13]. Singh et al. (2021) [13] confirmed that psychological factors like anxiety, daily hassles, negative life events, and coping strategies consistently predict injury occurrence across different sports populations.

Depression, anxiety, and stress constitute closely related yet distinct dimensions of mental health. Depression is typically associated with reduced motivation, impaired concentration, and psychomotor slowing; anxiety with heightened vigilance, threat sensitivity, and cognitive interference; and stress with sustained physiological and psychological strain in response to perceived demands [14]. Importantly, these constructs are strongly intercorrelated and often co-occur, forming complex mental health patterns rather than isolated symptoms. Neurocognitive and physiological evidence supports these functional distinctions, including impaired executive functioning in depression [14], attentional control disruption in anxiety [15], and sustained neuroendocrine activation under chronic stress [16]. Treating depression, anxiety, and stress as independent predictors may therefore obscure their joint effects and fail to capture meaningful configurations of psychological functioning relevant to injury occurrence.

Previous studies have reported associations between elevated depressive symptoms, anxiety, and perceived stress with increased injury occurrence across athletic and occupational populations. Proposed mechanisms include impaired attentional focus, altered risk perception, delayed reaction times, and maladaptive coping under pressure. However, empirical findings remain inconsistent, with some studies reporting weak, null, or context-dependent effects.

Importantly, most investigations rely on single-predictor models and implicitly assume linear relationships between psychological variables and injury outcomes. Such approaches neglect the possibility that injury occurrence may increase disproportionately at higher symptom levels and contribute to the limited variance explained by linear psychological predictors [4,17].

Moreover, treating depression, anxiety, and stress as isolated predictors may obscure clinically relevant synergies. For example, Gordon and Larivière [6] and Khoshakhlagh et al. [7] demonstrated that simultaneous exposure to psychosocial strain and physical workload markedly increased musculoskeletal symptom risk compared with isolated exposures. Similarly, Bayesian modeling approaches in sport populations have shown that joint elevations in stress, anxiety, and depressive symptoms improve injury occurrence classification beyond single-predictor models [18]. These findings indicate that cumulative psychological burden may be more informative than single dimensions considered in isolation.

Despite growing interest in psychological determinants of injury occurrence, several methodological limitations persist. Psychological variables are typically examined in isolation, modeled linearly, and evaluated using variable-centered approaches, despite their strong intercorrelations and frequent co-occurrence. As a result, little is known about whether the effect of one psychological factor depends on the level of another or whether injury occurrence follows non-linear or threshold patterns.

Given the modest explanatory power of individual predictors, complementary analytical perspectives are warranted. Dimensional approaches enable testing of non-linear and interaction effects, whereas person-centered approaches allow identification of distinct mental health profiles that may better reflect real-world patterns of psychological functioning and injury vulnerability. Evidence from network and mediation analyses demonstrates that accounting for indirect pathways, such as depression mediating stress-related effects, can materially alter inferred risk estimates [19].

Conversely, large prospective studies indicate that psychology-only linear prediction models show limited predictive performance, underscoring the need for integrative, multi-stage analytical strategies [20].

A review of the available literature indicates that no prior investigation has concurrently explored the non-linear functional relationship involving depression, anxiety, and stress in relation to injury occurrence, modeled their interaction effects within a multivariable framework, and identified mental health profiles to compare injury occurrence across psychologically homogeneous subgroups.

This study sought to examine how depression, anxiety, and stress are related to the outcome of interest and injury occurrence using an integrative analytical approach that accounts for non-linearity, symptom interaction, and mental health profiles. Specifically, we aimed to: (1) examine the functional form of the associations between depression, anxiety, and stress and injury occurrence, including potential non-linear effects; (2) assess the joint and interactive effects of depression, anxiety, and stress on injury occurrence, estimating adjusted odds ratios and predicted injury probabilities across meaningful combinations of psychological predictors; (3) identify mental health profiles based on depression, anxiety, and stress levels and compare injury occurrence across the identified profiles.

Based on contemporary stress–injury frameworks and prior empirical findings, it was hypothesized that higher levels of depression, anxiety, and stress would be associated with higher odds of injury occurrence, with potential non-linear and interactive effects amplifying the likelihood of injury at higher symptom levels. It was further hypothesized that distinct mental health profiles characterized by elevated cumulative distress would demonstrate higher injury prevalence compared with profiles reflecting lower psychological burden.

## 2. Materials and Methods

To ensure sufficient statistical power for analyses of sleep-related injury correlates, data from two separately recruited cohorts of university students were combined. Data collection took place between 2022 and 2023 and comprised objective assessments (anthropometry, body composition, and balance testing) alongside self-administered questionnaires addressing injury history, lifestyle factors (diet and physical activity), sleep parameters, mental health, quality of life, and socio-economic background. Although the present study forms part of an ongoing series of publications based on this dataset, it addresses a distinct research problem and is guided by original aims and specific research questions that have not been examined in previous reports.

### 2.1. Study Design

To enhance statistical precision, datasets from two successive annual recruitment waves conducted under the same protocol and ethical approval were integrated in a cross-sectional design. Injury occurrence (INJ) was treated as the dependent variable, whereas emotional states (ESs)—operationalized as depression (DPR), anxiety (ANX), and stress (STR)—were examined as key correlates. Preliminary findings indicated sex-related differences; therefore, subsequent analyses were stratified by sex rather than adjusted for it as a covariate. Within each sex, the analytical strategy addressed both the individual contributions and the combined (interactive) effects of ES components on injury occurrence, with additional attention given to the association between negative emotional state profiles and injury occurrence.

Baseline comparability between recruitment waves was assessed using standardized mean differences (SMDs). With the exception of age and injury prevalence, all SMD values were small (<0.30), indicating negligible imbalance in psychological and anthropometric variables. Although age differed statistically between waves, the absolute difference was less than one year and considered not clinically meaningful within this narrow young-adult range.

### 2.2. Ethics

The study protocol was reviewed and authorized by the Senate Research Ethics Committee of the Wroclaw University of Health and Sport Sciences (approval reference: 13/2022). Participation was voluntary, and electronic informed consent was secured from all individuals prior to data collection.

### 2.3. Sample Size

Sample size adequacy was evaluated using established heuristics for exploratory multivariate analyses (≥10 observations per predictor) and a complementary margin-of-error approach. Detailed procedures have been reported previously [21]. The final analytical sample comprised 418 students with complete data on key variables, which was considered sufficient for stable parameter estimation in the planned analyses.

### 2.4. Participants

Across the two cohorts, 454 students were initially enrolled. Eligibility criteria were defined as follows:

Inclusion criteria: (1) full-time university student aged 18–25 years; (2) regular participation in physical activity and attendance at on-site academic classes. Exclusion criteria: (1) current involvement in officially recognized university sports teams competing at inter-university or national level; (2) participation in structured elite or high-performance training programs exceeding standard curricular physical activity requirements; (3) exemption from mandatory university physical education classes for more than two consecutive weeks; (4) acute musculoskeletal injury within one month prior to assessment; (5) missing data in key outcome or exposure variables (injury occurrence or complete DASS-21 scale), or implausible/inconsistent questionnaire responses. Eligibility was verified during recruitment using standardized self-report screening items.

After applying the predefined inclusion criteria, 418 participants (199 men and 219 women) were retained for the final analyses and received complete data for the variables of interest. Participants were full-time university students aged 18–24 years and physically active. Individuals involved in elite or university-regulated competitive sports, those with diagnosed sleep, metabolic, or psychiatric disorders, night-shift workers, and cases with implausible questionnaire responses were excluded.

Prior to pooling, the two cohorts were compared with respect to demographic, anthropometric, emotional state, and exposure-related variables. No meaningful between-cohort differences were observed. Standardized mean differences (SMDs) were small across variables, supporting cohort comparability (Supplementary Table S1).

**Figure 1:**
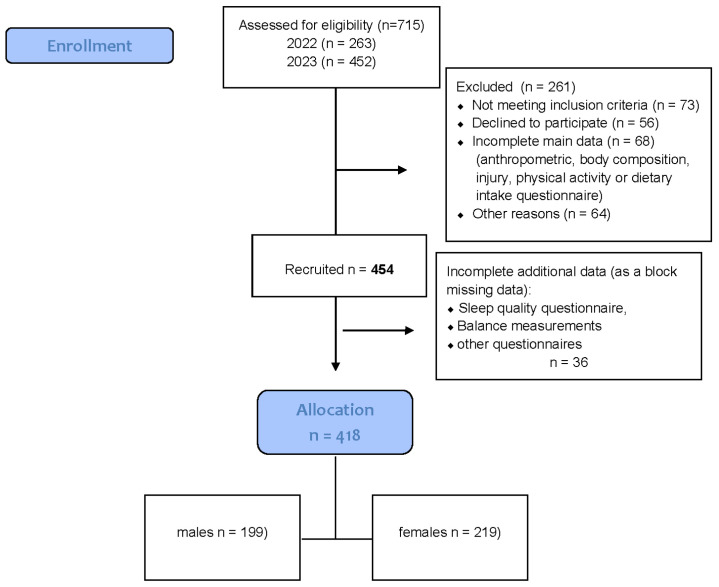
Flow diagram of the progress through the all phases of data collection.

The participant selection process is illustrated in Figure 1.

### 2.5. Anthropometric Measurements

All anthropometric assessments were conducted in standardized laboratory settings at the Biokinetics Research Laboratory, Wroclaw University of Health and Sport Sciences. Stature was recorded to the nearest 0.1 cm using a GPM anthropometer (GPM Instruments GmbH, Susten, Switzerland). Body mass and fat mass were determined with bioelectrical impedance analysis (InBody Co., Ltd., Seoul, Republic of Korea), with measurements obtained to an accuracy of 0.1 kg.

Based on these data, the Body Mass Index (BMI) was calculated according to the following formula:

$$\mathrm{BMI}\;=\;\frac{\mathrm{body}\;\mathrm{mass}\;[\mathrm{kg}]}{\mathrm{body}\;\mathrm{height}\;[m^{2}]}$$

### 2.6. Questionnaire Measurements

All questionnaire-derived variables were scored according to the original instrument protocols. Composite indicators used in modeling are described explicitly to allow replication, including item composition, scoring direction, and interpretation of a one-unit increase (or one SD after standardization).

#### 2.6.1. Injury Occurrence—Injury History Questionnaire (IHQ)

Musculoskeletal injury occurrence during the preceding 12 months was assessed using the standardized Injury History Questionnaire (IHQ), widely applied in epidemiological studies of physically active populations [22]. To facilitate regression analyses, injury data were operationalized as a binary outcome reflecting whether at least one injury had occurred (1) or not (0).

For the purpose of this study, injury was defined as a musculoskeletal complaint sustained during physical activity within the previous 12 months that resulted in pain accompanied by functional limitation or modification of usual activity. The definition did not require medical consultation or formal time-loss from sport or exercise. Both acute traumatic injuries and overuse injuries were included. Minor transient complaints that did not affect participation or functional performance were not classified as injuries.

#### 2.6.2. Negative Emotional States—Depression, Anxiety, and Stress Scale (DASS21)

Emotional distress was evaluated using the 21-item version of the Depression, Anxiety, and Stress Scale (DASS-21), a validated self-administered questionnaire assessing three dimensions: depression (DPR), anxiety (ANX), and stress (STR) [23,24,25]. Internal consistency in the present sample was satisfactory, with Cronbach’s α coefficients of 0.910 for depression, 0.831 for anxiety, and 0.936 for stress.

The instrument was chosen because of its concise format, robust psychometric performance, and appropriateness for epidemiological research in young adult samples.

#### 2.6.3. Training Weekly Load (TWL) and Physical Activity Experience (EXP)

Training weekly load (TWL) was estimated from self-reported training frequency (sessions per week) and average session duration (hours) and expressed as total training time per week (h·week^−1^). This time-based indicator was used as a pragmatic measure of habitual training exposure in a cross-sectional, questionnaire-based student sample.

Training experience (EXP) was defined as the self-reported number of years of regular engagement in physical activity, regardless of sport discipline.

### 2.7. Handling and Imputation of Missing Data

Missing data were minimal and consistent with the MCAR assumption based on logistic regression diagnostics. Multiple imputation by chained equations (MICE) was performed in R (20 imputations), and estimates were pooled using Rubin’s rules.

### 2.8. Statistics

Descriptive statistics are presented as means and standard deviations as well as medians and interquartile ranges for continuous variables and counts and percentages for categorical variables.

Injury occurrence (INJ) was treated as a binary outcome variable. All main analyses were conducted using logistic regression models. As logistic regression does not require normally distributed predictors, no formal tests of normality were applied to depression, anxiety, stress, training weekly load (TWL), or experience (EXP) variables.

Training weekly load (h·week^−1^) and years of physical activity experience were considered potential confounding variables, as both may influence exposure to injury as well as emotional distress. TWL and EXP were treated as confounders rather than mediators, as the study aimed to estimate the association between emotional states and injury occurrence independent of exposure and training history. Therefore, all regression analyses were adjusted for TWL and EXP.

To examine the functional form of the associations between emotional states and injury occurrence, a series of competing logistic regression models was fitted, including linear terms, polynomial terms, and natural cubic splines for depression, anxiety, and stress scores. All models in this step included TWL and EXP as covariates. Model fit was evaluated using likelihood ratio tests and information criteria (AIC and BIC), and predicted probability curves were visualized to illustrate non-linear injury occurrence probability patterns.

Subsequently, multivariable logistic regression models were constructed to estimate the independent effects of depression, anxiety, and stress on injury occurrence while adjusting for TWL and EXP. Interaction terms between emotional state variables were included to assess potential synergistic effects. Continuous predictors were mean-centered prior to inclusion of interaction terms to reduce multicollinearity. Results are reported as odds ratios (ORs) with 95% confidence intervals (CIs). Correlations between depression, anxiety, and stress scores were examined, and variance inflation factors (VIFs) were calculated to assess multicollinearity. Results indicated acceptable levels of intercorrelation and no evidence of problematic collinearity (Supplementary Tables S2 and S3).

Profile analysis based on mixed-effects modeling was performed to examine whether the configuration of depression (DPR), anxiety (ANX), and stress (STR) differed according to injury occurrence. Prior to analysis, component scores were standardized to ensure comparability across scales. The profile analysis framework evaluated three aspects: flatness (whether component means differ within individuals), level (overall profile elevation between injury groups), and parallelism (whether profile shapes differ across injury occurrence). Training weekly load (TWL) and physical activity experience (EXP) were not included as components of the profile itself but were incorporated as covariates in the mixed-effects models to adjust for potential exposure-related confounding. Type III tests with Satterthwaite’s approximation were used to evaluate fixed effects. This approach allows examination of multivariate configuration differences across emotional state components without assuming class structure or mixture distribution.

To enhance transparency and reproducibility, all modeling steps were predefined and implemented consistently across sex-stratified analyses. Multivariable models were treated as the primary inferential framework, while additional procedures (functional-form checks and profile analysis) were used to assess robustness and to provide complementary descriptive insight rather than to redefine the main hypothesis-testing models.

To assess potential multicollinearity among depression, anxiety, and stress scores, variance inflation factors (VIFs) and tolerance statistics were calculated. All VIF values were below 5.0, indicating no evidence of problematic multicollinearity.

Data analyses were carried out with the use of Statistica 14.0 (TIBCO Software Inc., Palo Alto, CA, USA) as well as RStudio (v2025.09.1 + 401). The significance threshold was set at *p* < 0.05.

### 2.9. AI Transparency Statement

Generative AI tools were used in accordance with COPE and MDPI transparency guidelines and were limited to preparatory, technical, and editorial support. AI-assisted platforms supported literature identification, methodological orientation, and manuscript preparation but did not influence the study design, data collection, statistical modeling strategy, or interpretation of results.

Chat Academia (v.1.0, 2025) and Elicit (v.2.0, 2025) were used to refine research questions and facilitate literature searches, while SciSpace (2025) assisted in organizing reviewed studies and clarifying methodological terminology. In selected instances, draft code fragments were generated using Julius.ai (2025) or ChatGPT (OpenAI, GPT-4.1, 2025) for technical support (e.g., resolving syntax issues or clarifying R documentation). All AI-generated material, including draft code and language suggestions, was manually reviewed, revised where necessary, and independently verified by re-running analyses within the R environment.

The author assumes full responsibility for the integrity, accuracy, and final content of the manuscript.

## 3. Results

### 3.1. Participant Characteristics

Results are presented primarily as associations derived from cross-sectional models. Accordingly, interpretations are restricted to statistical relationships rather than causal effects.

Participant characteristics at study entry are detailed in Table 1. Quantitative variables are presented as the mean ± SD, accompanied by 95% CIs. Significant sex-based disparities were identified in body size and composition measures, with men exhibiting higher values for each anthropometric parameter examined (all *p* < 0.05). There were no significant differences between men and females in the components of negative emotional states or training weekly load, whereas the sexes differed significantly in training experience (*p* < 0.001).

The study population consisted of 199 men and 219 women (47.6% and 52.4%, respectively). Injury was reported more frequently by males (56.8%) than by females (46.6%). Correspondingly, the odds of injury occurrence were 34% lower in females compared with males (χ = 4.36; *p* = 0.037; φ = −0.10; OR = 0.66).

### 3.2. Comparison of Logistic Model Functional Forms

To characterize the functional form of the associations between negative emotional states and injury occurrence, a series of nested logistic regression models with increasing polynomial complexity were fitted separately for depression (DPR), anxiety (ANX), and stress (STR). For each independent association, linear, quadratic, and cubic specifications were compared. All models were adjusted for training experience (EXP) and training weekly load (TWL) (Table 2). Because depression, anxiety, and stress levels did not differ between males and females, analyses evaluating the functional form of associations were conducted in the pooled sample. Sex-specific analyses were subsequently performed to account for known differences in injury occurrence.

Model comparisons based on the AIC, the BIC, and likelihood ratio tests indicated no evidence that quadratic or cubic terms improved model fit for DPR or STR. In both cases, neither the quadratic nor the cubic specification provided a significantly better fit than the linear model, and information criteria consistently favored the linear form.

For ANX, the cubic specification yielded the lowest AIC and showed a statistically significant improvement over the quadratic specification (LR test *p* = 0.010); however, this improvement was not supported by BIC and primarily reflected increased model complexity without a clear or clinically interpretable functional pattern. The quadratic model did not outperform the linear specification.

Based on model fit and interpretability criteria, the linear specification was deemed the most appropriate functional form for representing associations between negative emotional states and injury occurrence and was therefore used in the remaining analyses in accordance with the principle of parsimony and the lack of consistent support across information criteria.

To enhance interpretability of model comparisons, predicted probability curves for linear, quadratic, and cubic specifications of anxiety were visualized within the 5th–95th percentile range of observed values (Supplementary Figure S1). The cubic model demonstrated local curvature; however, these deviations were modest within the central data range and did not reflect a clinically meaningful threshold pattern. In contrast, the linear specification provided a stable and interpretable representation across the observed distribution.

### 3.3. Sex-Stratified Multivariable Logistic Regression Models (Adjusted for EXP and TWL)

To evaluate whether combinations of negative emotional states exert synergistic effects on injury occurrence, sex-stratified multivariable logistic regression models were fitted separately for females and men. Base models including depression (DPR), anxiety (ANX), and stress (STR) as simultaneous variables associated with injury occurrence were compared with extended models additionally incorporating all two-way interaction terms between emotional state components (DPR × ANX, DPR × STR, ANX × STR). All models were adjusted for training experience (EXP) and training weekly load (TWL). Results are presented in Table 3.

As shown in Table 3, the inclusion of interaction terms resulted in a consistent deterioration of model fit in both sexes. For females, both the AIC and the BIC increased after adding interaction terms, and the likelihood ratio test did not indicate a significant improvement over the base model (LR *p* = 0.595). Similarly, in men, interaction models were characterized by higher AIC and BIC values and a non-significant likelihood ratio test (LR *p* = 0.362). Accordingly, the base multivariable models were retained as the most parsimonious and appropriate specifications for both sexes.

**Figure 2:**
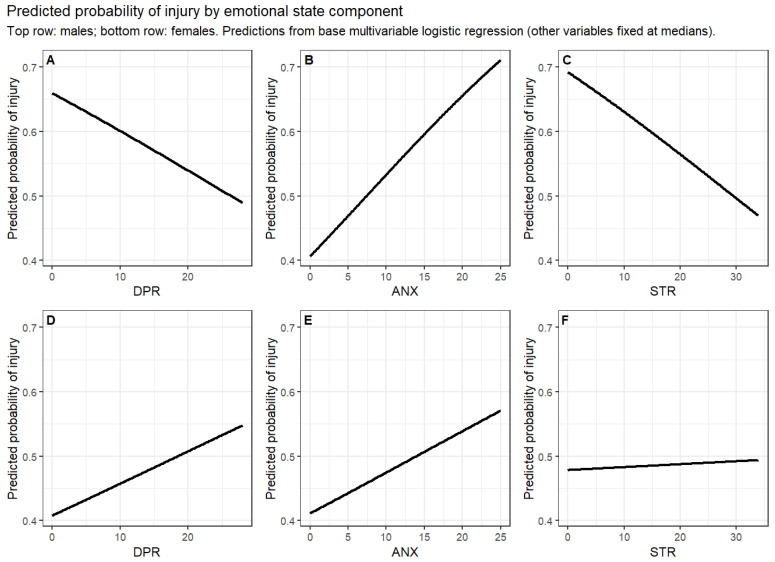
Predicted probability of injury occurrence as a function of negative emotional state components, stratified by sex. Panels (**A**–**C**) present predicted injury probability in men as a function of depression (DPR), anxiety (ANX), and stress (STR), respectively, while panels (**D**–**F**) show the corresponding relationships in females. Predictions were derived from sex-stratified base multivariable logistic regression models including depression, anxiety, and stress simultaneously and adjusted for training experience (EXP) and training weekly load (TWL). For each panel, the focal variable is shown across its observed range, whereas the remaining emotional state components and covariates were fixed at their median values.

In the final base models, in males, anxiety showed a small but statistically significant positive association with injury occurrence, whereas depression and stress were not significant variables associated with injury occurrence. To facilitate clinical interpretation, the odds ratio for anxiety in males (OR = 1.05 per one-point increase) corresponds to an approximate 28% increase in the odds of injury occurrence per 5-point increase (1.05^5^ ≈ 1.28) and a 63% increase per 10-point increase (1.05^10^ ≈ 1.63), assuming linearity of the log-odds. Despite statistical significance, the absolute magnitude of this association remains modest within the observed score range. Training experience and training weekly load were not significantly associated with injury occurrence in either sex. In females, none of the negative emotional state components were significantly associated with injury occurrence (Table 4, Figure 2).

Overall, these findings indicate that the effects of depression, anxiety, and stress on injury occurrence are predominantly additive rather than interactive, with no statistically detectable interaction or synergistic effects in this sample.

Detailed model diagnostics (Nagelkerke R^2^, AUC, calibration, and multicollinearity indices) are provided in Supplementary Table S4.

### 3.4. Sex-Stratified Profile Analysis of Negative Emotional States by Injury Occurrence

Profile analysis was used to examine whether the pattern (flatness), overall level (level), and shape similarity (parallelism) of negative emotional state components—depression, anxiety, and stress—differed according to injury occurrence. Flatness tests whether component scores differ in magnitude within a profile, level assesses differences in the overall height of profiles between groups, and parallelism evaluates whether profile shapes differ across groups.

**Figure 3:**
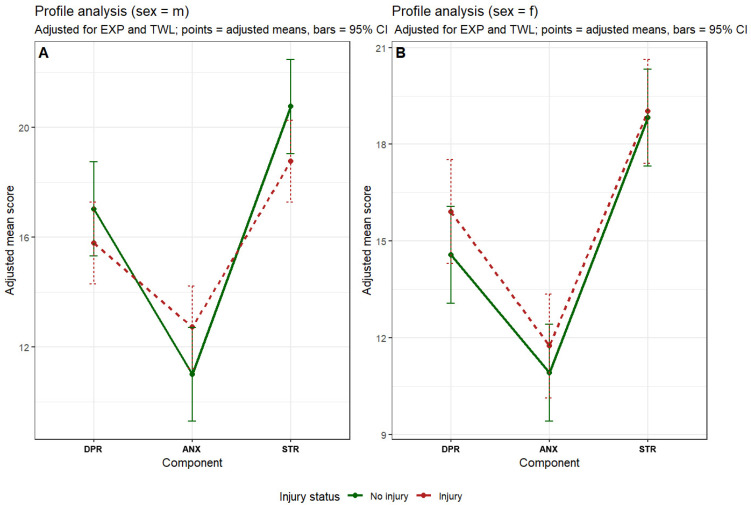
Sex-stratified profile analysis of negative emotional state components by injury occurrence. Panel (**A**) presents the adjusted profiles of depression (DPR), anxiety (ANX), and stress (STR) in men, whereas Panel (**B**) shows the corresponding profiles in females. Profiles were estimated using mixed-effects models adjusted for training experience (EXP) and training weekly load (TWL). Green lines indicate participants without injury, and red lines indicate injured participants. Points represent adjusted mean values, and error bars denote 95% confidence intervals.

As shown in Table 5 and Figure 3, profiles were non-flat in both men and women, indicating significant differences in magnitude between depression, anxiety, and stress components (*p* < 0.001 for both sexes). However, the overall profile level did not differ between injured and non-injured participants in either sex, suggesting that injury occurrence was not associated with a global elevation of negative emotional states. With respect to profile shape, no evidence of non-parallel profiles was observed in women, indicating highly similar component patterns between injured and non-injured groups. In men, a borderline trend toward non-parallel profiles was detected, driven by relatively higher anxiety scores among injured participants; however, this effect did not reach statistical significance and should be interpreted cautiously.

Overall, the combined evidence from profile analysis indicates that although depression, anxiety, and stress represent distinct emotional dimensions, injury occurrence is not associated with a distinct emotional profile configuration in either sex, and any potential deviations in profile shape appear limited and sex-specific.

## 4. Discussion

This study investigated the associations between depression, anxiety, stress, and injury occurrence in physically active young adults while accounting for training exposure and experience. Across all analyses, linear models provided the most parsimonious description of these relationships, with no statistically detectable non-linear or synergistic effects within the available sample size. Anxiety showed a small but statistically significant association with injury occurrence in males, whereas no significant associations were observed in females. Interaction models and profile analysis did not identify distinct emotional configurations differentiating injured from non-injured participants. Overall, negative emotional states appear to contribute modestly and independently to injury occurrence, with limited explanatory value in this population.

Although the association between anxiety and injury in males reached statistical significance (OR = 1.05), its magnitude was modest. A one-point increase in anxiety score corresponded to approximately a 5% increase in injury odds, translating to roughly a 1-percentage-point increase in absolute injury probability at the observed baseline risk. Across a clinically meaningful range of approximately five points (≈one standard deviation), the estimated injury probability increased from about 57% to approximately 63–64%, representing a 6–7-percentage-point difference. While detectable at the population level, this effect size suggests limited standalone predictive utility in individual injury screening. From a practical standpoint, anxiety should therefore be interpreted as one contributory factor within a broader multifactorial injury framework rather than as a dominant clinical determinant.

Across university student samples, psychological distress shows generally modest associations with injury occurrence. Among negative emotional states, anxiety most consistently emerges as a clearer correlate than depression or stress, although findings regarding sex differences remain mixed. Several studies further suggest that combined or cumulative indicators of psychological distress may outperform single measures when explaining injury occurrence, particularly in longitudinal designs.

Galambos et al. (2005) [4] demonstrated that mood states and perceived stress had statistically significant but modest predictive utility for injury. In a subset of 233 initially uninjured elite athletes (116 women, 117 men), five mood dimensions each explained approximately 6–7% of the variance in retrospective orthopedic injury incidents, while overall injury occurrence classification accuracy based on mood and stress measures reached only 39%. Similarly, Lavallée and Flint (1996) [26] reported moderate associations between negative mood states and injury outcomes in a smaller sample of 55 male Canadian varsity athletes, primarily football and rugby players. Correlations between tension/anxiety and injury frequency or severity ranged from r = 0.29 to 0.44, indicating small-to-moderate continuous relationships. In the same varsity athlete sample, competitive anxiety and tension/anxiety mood states showed stronger associations with injury frequency and severity than other mood dimensions, including depression-related scales. Consistent with this pattern, a cross-sectional study of 187 football and futsal players reported that injured male athletes exhibited significantly higher anxiety scores than non-injured males, suggesting that anxiety distinguished injury occurrence within this subgroup when assessed using the DASS-21 questionnaire [27].

A recent systematic review examining associations between musculoskeletal injuries and depressive symptoms found that female athletes tended to report higher depressive symptoms following injury than males, with several studies indicating sex-related differences in psychological responses to injury across diverse athletic populations [28]. In line with these observations, the football and futsal study described above identified sex-specific patterns: injured males reported higher anxiety than non-injured males, whereas among females, higher stress levels were observed in non-injured players compared with injured counterparts [27]. In contrast, Galambos et al. (2005) [4] reported no sex differences in the relationships between mood, perceived stress, and injury outcomes in their large elite-athlete screening dataset, which included 845 assessments and subset analyses. Moreover, Andreu et al. (2014) [29] identified personality-based vulnerability profiles in 453 competitive athletes (285 men, 168 women) and found that athletes classified as psychologically “vulnerable” did not consistently exhibit higher injury rates. In some sex- and sport-specific subgroups, vulnerable profiles were even associated with fewer or less severe injuries, producing patterns opposite to those commonly hypothesized.

Evidence suggests that cumulative psychological burden may be more informative than individual emotional components. Results showed that combined mood and perceived stress measures provided greater predictive utility than single scales and explicitly recommended incorporating broader allostatic indicators into injury occurrence models, given the modest variance explained by psychometric measures alone [4]. Prospective season-long study of a collegiate football team further demonstrated that negative life stress predicted injury occurrence and that this relationship was moderated by multiple psychosocial factors, including sport anxiety, coping resources, social support, athletic identity, and playing status, underscoring the importance of cumulative and interacting psychosocial influences [30]. On the other hand, the use of a probabilistic model extended traditional stress–injury frameworks by jointly incorporating stress, anxiety, and depression within a Bayesian network, showing that combined negative psychological features captured additional structure relevant to injury occurrence beyond linear single-variable approaches [27].

Most of the above-mentioned studies support graded or approximately linear associations between psychological distress and injury occurrence. Correlational analyses demonstrated continuous relationships between anxiety-related mood states and injury frequency or severity [26]. Similarly, individual mood dimensions explained small but additive proportions of variance in injury outcomes, consistent with linear contributions of psychological factors [4]. However, some of the results are contradictory and suggest non-linear or threshold-like relationships. Haghshenas and Molavi (2008) [31] applied discriminant analysis in a sample of 169 male athlete students and identified a questionnaire cut-off score (>83) that differentiated higher- from lower-risk athletes, implying the presence of a potential psychological threshold associated with injury risk occurrence in that population. In addition, the Bayesian network approach employed by Zafra et al. (2022) [32] revealed non-linear and conditional dependencies among stress, anxiety, depression, and injury, indicating that probabilistic and non-linear models may better reflect the complexity of psychosocial injury occurrence in certain contexts.

Cross-sectional screening studies in elite athletes and team-sport players consistently report graded associations between psychological distress and injury occurrence but are inherently limited in establishing temporal or dose–response relationships [4,27]. In contrast, prospective and longitudinal designs, including collegiate football season studies and trail running cohorts, provide stronger evidence that elevated pre-injury stress or negative mood predicts subsequent injury occurrence, with cumulative exposure and moderating factors shaping trajectories of injury occurrence over time [28,30,33].

Several studies have treated depression, anxiety, and stress as largely independent predictors. Haghshenas and Molavi analyzed cognitive and somatic anxiety as well as self-confidence dimensions separately when classifying injured versus non-injured athletes [31]. Likewise, correlational studies frequently report distinct associations for anxiety, depression, and other mood subscales without modeling interactions explicitly [26]. Conversely, interaction and moderation effects have been documented in prospective research. Effect of negative life stress on injury was contingent upon multiple psychosocial moderators, highlighting the interdependence of psychological constructs in shaping injury occurrence [30]. Galambos et al. also suggested joint effects of mood and stress, although formal statistical interactions were not tested [4]. From an allostatic load perspective, these authors explicitly advocated for integrating physiological stress indicators with psychometric measures, as psychological variables alone accounted for only a limited proportion of injury variance. Similarly, Bayesian framework operationalized cumulative psychological burden by modeling combinations of stress, anxiety, and depression [32]. The systematic review by Marconcin et al. further supported bidirectional and cumulative relationships between musculoskeletal injuries and depressive symptoms, reinforcing the relevance of psychosocial burden across injury and recovery processes [28].

Several studies have applied profile- or cluster-based methods to identify psychological subgroups associated with injury occurrence. Gajardo-Burgos et al. used Gaussian mixture modeling in a prospective cohort of 202 trail runners and identified distinct psychological profiles linked to running-related injuries [33]. Madigan et al. (2018) applied latent profile analysis prospectively to classify athletes according to personal and interpersonal correlates of overuse injuries, identifying subgroups with differential injury susceptibility [34]. Prieto Andreu et al. similarly described personality vulnerability profiles in competitive athletes and examined injury frequency and severity across profile groups [29]. While these studies provide evidence for distinct psychological profiles associated with injury outcomes, other findings suggest limited discriminative power. In particular, low classification accuracy for injury occurrence based solely on psychological screening data in elite athletes, indicating that psychometric profiles alone may be insufficient for reliable injury occurrence stratification in heterogeneous athletic populations has been previously reported [4].

In summary, the present findings indicate that negative emotional states are at most modest and largely independent contributors to injury occurrence in physically active young adults, with anxiety emerging as a weak but consistent signal in males, while no stable emotional profiles or synergistic effects were identified.

Importantly, the cross-sectional nature of the present study requires careful interpretation of the directionality of observed associations. Emotional distress may function either as a predisposing vulnerability factor increasing injury susceptibility or as a psychological response to prior injury experiences. While theoretical stress–injury frameworks conceptualize anxiety and stress as mechanisms that may disrupt attentional focus, impair motor coordination, or alter risk perception, the reverse pathway is equally plausible. Injury experiences may elevate anxiety through uncertainty regarding return-to-play, performance capability, or social evaluation. This mechanism may be particularly relevant in males, where sport-related identity and performance expectations are often strongly internalized. In such cases, anxiety may reflect injury-related hypervigilance, fear of re-injury, or rehabilitation-related stress rather than a direct etiological contributor to injury occurrence. Consequently, the present findings should be interpreted as associative and hypothesis-generating rather than causal, and longitudinal designs are necessary to disentangle bidirectional dynamics between emotional distress and injury occurrence.

This study has several important limitations. Most notably, its cross-sectional design prevents conclusions regarding causality and does not permit determination of the temporal order between emotional distress and injury occurrence. Emotional states may act both as antecedents and as consequences of injury experiences. Although bidirectional mechanisms were discussed above, temporal sequencing cannot be empirically established within the present design. Because both injury data and emotional variables were obtained through self-report, the findings may be influenced by reporting bias. In particular, retrospective injury assessment can be susceptible to recall error, and the shared measurement format may inflate observed associations. Third, the study relied on questionnaire-based indicators of emotional distress and did not include physiological or behavioral stress markers; therefore, cumulative or allostatic load could not be directly assessed. Additionally, individuals with diagnosed psychiatric disorders were excluded from participation to improve internal validity and reduce clinical heterogeneity. While this approach strengthens interpretability within a non-clinical population, it may have restricted the upper range and overall variability of emotional distress scores. Such range restriction could attenuate statistical power to detect non-linear, threshold-like, or high-risk effects, particularly at extreme levels of anxiety, depression, or stress. Consequently, the absence of identified non-linear associations should be interpreted in the context of a relatively homogeneous and psychologically healthy sample. Fourth, although analyses were adjusted for training exposure and experience, unmeasured confounders such as sleep quality, recovery strategies, biomechanical factors, or acute workload fluctuations may have influenced injury occurrence. In particular, factors such as prior injury history and severity, sleep disturbances, inadequate recovery behaviors, or short-term workload spikes may plausibly be associated both with elevated anxiety levels and with increased injury susceptibility. The absence of these variables raises the possibility of residual confounding, whereby the observed anxiety–injury association in males may partially reflect shared variance with unmeasured physiological or behavioral risk factors. Consequently, the independent contribution of anxiety should be interpreted cautiously, as its effect size may be overestimated or attenuated depending on the direction and magnitude of unmeasured confounding influences. Moreover, training weekly load (TWL) was operationalized solely as total time spent in physical activity and did not incorporate intensity, modality, or short-term workload variability. This time-based measure may incompletely capture true biomechanical and physiological exposure. Such non-differential exposure misclassification likely biases associations toward the null, potentially attenuating observed effect sizes. Therefore, the modest magnitude of associations should be interpreted in light of possible exposure measurement limitations. Additionally, injury occurrence was operationalized as a binary outcome without differentiation by anatomical location, severity, mechanism (acute vs. overuse), or time-loss characteristics. The lack of injury-type specification limits the ability to determine whether psychological distress may be differentially associated with particular injury categories. Moreover, injury heterogeneity may have attenuated or obscured potential psychological associations. It is plausible that anxiety, stress, or depressive symptoms relate differently to acute traumatic injuries compared with overuse injuries, or to injuries of varying severity. Aggregating heterogeneous injury types into a single binary outcome may therefore have reduced sensitivity to detect mechanism-specific psychological patterns. Future research incorporating more granular injury classification may provide deeper insight into potential mechanism-specific psychological patterns of injury occurrence. Next, the profile analysis was included as a complementary, data-driven description of behavioral/psychological patterns and should not be interpreted as causal segmentation; conclusions remain anchored in the primary regression/SEM findings. Finally, although the overall sample size was sufficient for detecting main effects in multivariable logistic regression models, interaction and profile-based analyses generally require substantially greater statistical power to identify small synergistic or shape-related effects. The borderline parallelism result observed in males (*p* = 0.055) may therefore reflect limited power to detect subtle deviations in profile configuration rather than definitive evidence of absence of effect. Small interaction effects are inherently more difficult to detect than main effects, particularly in sex-stratified analyses. Consequently, the absence of statistically significant synergistic findings in this sample should not be interpreted as definitive evidence of absence. The composition of the sample represents an additional constraint. As participants were physically active university students, the applicability of the findings to elite sport settings, clinical cohorts, or high-risk occupational groups may be restricted.

## 5. Conclusions

In physically active young adults, negative emotional states show limited and sex-specific associations with injury occurrence. Anxiety was weakly associated with injury occurrence in males, whereas depression and stress were not significant independent correlates in either sex. The absence of non-linear effects, interaction effects, and distinct emotional profiles indicates that emotional states contribute to injury occurrence in an additive and modest manner rather than through synergistic or pattern-based mechanisms.

These findings suggest that psychological screening based solely on depression, anxiety, and stress may have limited utility for injury occurrence stratification in heterogeneous, non-elite populations. Prospective, longitudinal approaches would allow clearer insight into causal pathways and should incorporate psychological constructs alongside biomechanical, behavioral, and exposure-based determinants to more comprehensively model injury occurrence.

## Figures and Tables

**Table 1:** Baseline characteristics of the study participants (n = 418).

Variable			Males			Females			
	Mean	95% CI Lower	95% CI Upper	SD	Mean	95% CI Lower	95% CI Upper	SD	*p*
Age [y]	20.73	20.61	20.85	0.85	20.56	20.46	20.65	0.74	**0.025**
Body height [cm]	182.19	181.20	183.18	7.10	168.17	167.37	168.97	6.01	**<0.001**
Body weight [kg]	79.63	78.25	81.01	9.87	60.86	59.65	62.06	9.05	**<0.001**
BMI [kg/m^2^]	23.97	23.62	24.32	2.48	21.49	21.12	21.85	2.71	**<0.001**
Depression [scores]	16.3	15.2	17.5	8.3	15.2	14.1	16.3	8.1	0.162
Anxiety [scores]	12.0	11.1	12.8	5.9	11.3	10.5	12.1	5.9	0.243
Stress [scores]	19.6	18.3	21.0	9.7	18.9	17.6	20.3	10.2	0.470
Experience [years]	3.6	3.4	3.8	1.3	3.1	2.9	3.3	1.5	**<0.001**
Training weekly load [h·week^−1^]	6.1	5.6	6.7	3.9	5.7	5.1	6.2	3.8	0.201

Footnote: CI—Confidence; SD—Standard Deviation; BMI—body mass index; statistical significance is marked in bold font.

**Table 2:** Comparison of logistic regression models with alternative functional forms of the association between negative emotional states and injury occurrence (adjusted for EXP and TWL).

Variable	Model	AIC	BIC	LR Test (*p*)
	Linear	584.97	601.11	–
Depression (DPR)	Quadratic	586.10	606.28	0.352
	Cubic	587.23	611.44	0.351
	Linear	579.96	596.11	–
Anxiety (ANX)	Quadratic	581.45	601.62	0.471
	Cubic	576.86	601.08	0.010
	Linear	584.51	600.65	–
Stress (STR)	Quadratic	586.51	606.68	0.988
	Cubic	587.76	611.97	0.387

Footnote: AIC—Akaike Information Criterion; BIC—Bayesian Information Criterion; LR test—likelihood ratio test.

**Table 3:** Comparison of base multivariable logistic regression models and interaction models predicting injury occurrence, stratified by sex. Models adjusted for training experience (EXP) and training weekly load (TWL). Lower AIC and BIC indicate better model fit.

Sex	Model Specification	AIC	BIC	LR Test (*p*)
Female	Base model (DPR + ANX + STR)	309.0	329.4	–
Female	Interaction model (DPR × ANX, DPR × STR, ANX × STR)	313.1	343.6	0.595
Male	Base model (DPR + ANX + STR)	274.8	294.6	–
Male	Interaction model (DPR × ANX, DPR × STR, ANX × STR)	277.6	307.3	0.362

Footnote: DPR—depression; ANX—anxiety; STR—stress; AIC—Akaike Information Criterion; BIC—Bayesian Information Criterion; LR test—likelihood ratio test.

**Table 4:** Sex-stratified multivariable logistic regression models predicting injury occurrence. Base multivariable logistic regression models including depression (DPR), anxiety (ANX), and stress (STR) as simultaneous variables associated with injury occurrence and adjusted for training experience (EXP) and training weekly load (TWL). Values are odds ratios (ORs) with 95% confidence intervals (CIs).

Sex	Variable	OR	95% CI	*p*-Value
Female	DPR	1.02	0.99–1.06	0.238
	ANX	1.03	0.98–1.08	0.278
	STR	1.00	0.98–1.03	0.894
	EXP	1.16	0.96–1.39	0.131
	TWL	0.96	0.89–1.03	0.248
Male	DPR	0.98	0.94–1.01	0.172
	ANX	1.05	1.00–1.11	**0.043**
	STR	0.97	0.94–1.00	0.083
	EXP	1.02	0.81–1.27	0.889
	TWL	1.04	0.97–1.12	0.300

Footnote: DPR—depression; ANX—anxiety; STR—stress; EXP—training experience, TWL—training weekly load; OR—odds ratio; CI—confidence interval. Statistical significance is presented in bold font.

**Table 5:** Sex-stratified profile analysis of negative emotional state components by injury occurrence. Profile analysis based on mixed-effects models adjusted for training experience (EXP) and training weekly load (TWL). *p*-values correspond to Type III tests with Satterthwaite’s approximation.

Sex	Profile Test	Interpretation	*p*
Male	Flatness (component)	Components differ in magnitude (DPR ≠ ANX ≠ STR)	<0.001
Male	Level (injury)	Overall profile level does not differ by injury occurrence	0.444
Male	Parallelism (component × injury)	Trend toward non-parallel profiles	0.055
Female	Flatness (component)	Components differ in magnitude (DPR ≠ ANX ≠ STR)	<0.001
Female	Level (injury)	Overall profile level does not differ by injury occurrence	0.223
Female	Parallelism (component × injury)	Profiles are parallel	0.763

## Data Availability

The data presented in this study are available upon request from the author.

## Supplementary Materials

The following supporting information can be downloaded at https://www.mdpi.com/article/10.3390/jcm15051822/s1. Figure S1: Predicted probability of injury occurrence as a function of anxiety score under linear, quadratic, and cubic model specifications. Curves are shown within the 5th–95th percentile range of observed anxiety values to reduce the influence of extreme observations. All models were adjusted for training weekly load and training experience.; Table S1: Baseline comparability between recruitment waves (stu22 vs. stu23) assessed using standardized mean differences (SMD). Values are presented as mean ± SD for continuous variables and as proportions for injury occurrence. SMD values quantify between-cohort imbalance independent of sample size (values closer to 0 indicate better balance); Table S2: Pearson correlation matrix for depression, anxiety, and stress (DASS-21 subscales); Table S3: Multicollinearity diagnostics (VIF and tolerance) for predictors used in sex-stratified multivariable models; Table S4: Model diagnostics for sex-stratified multivariable logistic regression models predicting injury occurrence (adjusted for DPR, ANX, STR, EXP, and TWL).
