# Supplementary material for: Associations Between Emotional Distress and Injury Occurrence in Physically Active Students
Source: J Clin Med. 2026 Feb 27;15(5):1822. doi: 10.3390/jcm15051822 (PMC12986016; doi:10.3390/jcm15051822)
Supplement: Supplementary file 1 [file jcm-15-01822-s001.zip › Figure S1_Prdicted probability of injury occurence.pdf]

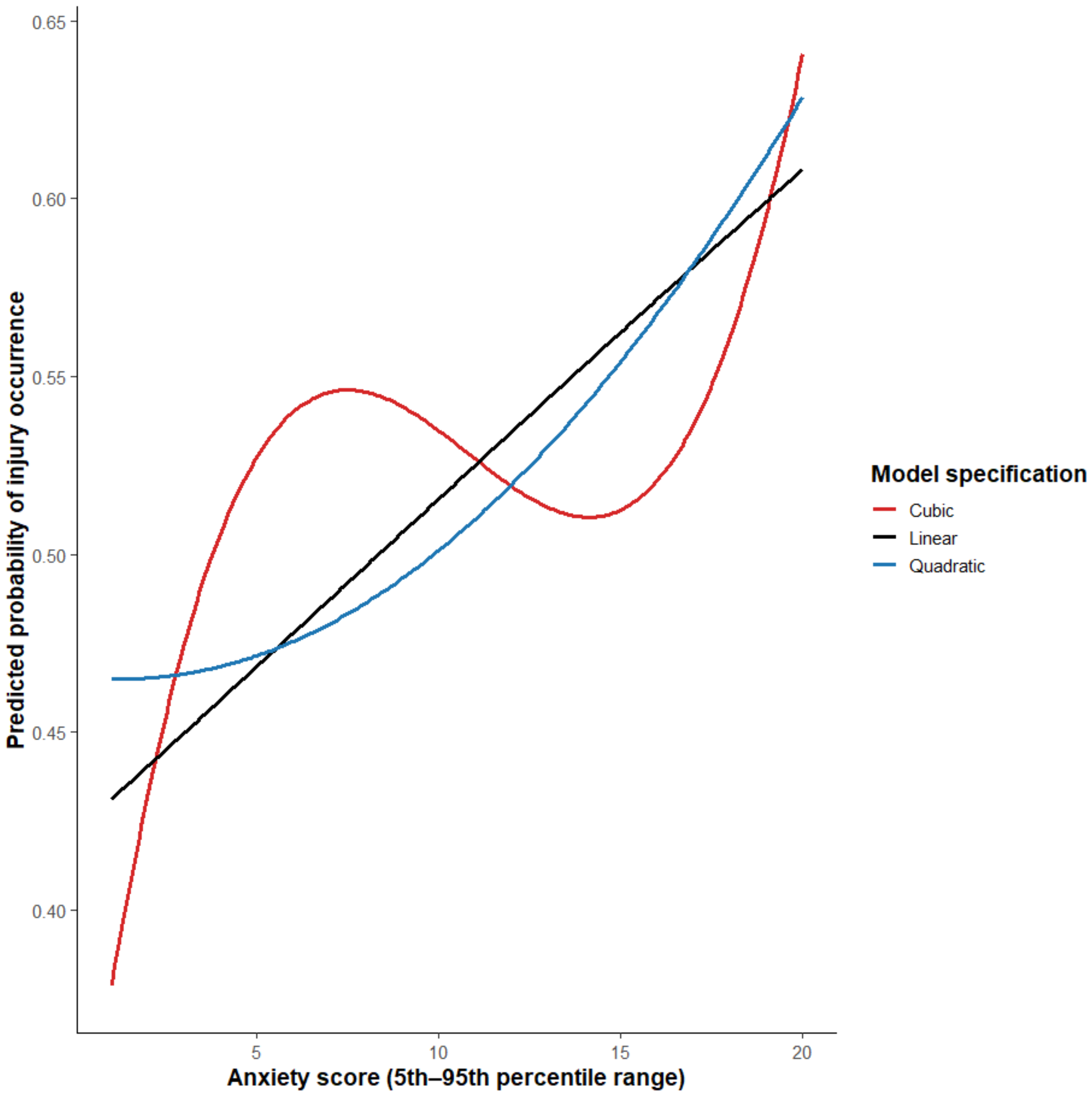

Figure S1. Predicted probability of injury occurrence as a function of anxiety score under linear, quadratic, and cubic model specifications. Curves are shown within the 5th–95th percentile range of observed anxiety values to reduce the influence of extreme observations. All models were adjusted for training weekly load and training experience.
