# Supplementary material for: Associations Between Emotional Distress and Injury Occurrence in Physically Active Students
Source: J Clin Med. 2026 Feb 27;15(5):1822. doi: 10.3390/jcm15051822 (PMC12986016; doi:10.3390/jcm15051822)
Supplement: Supplementary file 1 [file jcm-15-01822-s001.zip › Table S1_Baseline comparability between recruitment waves.pdf]

Table S1. Baseline comparability between recruitment waves (stu22 vs. stu23) assessed using standardized mean differences (SMD). Values are presented as mean ± SD for continuous variables and as proportions for injury occurrence. SMD values quantify between-cohort imbalance independent of sample size (values closer to 0 indicate better balance).

Variable	stu22 (n=200)	stu23 (n=218)	SMD
Body height (cm)	174.60 ± 9.68	175.07 ± 9.53	0.049
Body weight (kg)	70.39 ± 13.83	69.28 ± 12.82	-0.083
BMI (kg/m²)	22.89 ± 2.89	22.46 ± 2.86	-0.152
Depression (DASS-21 score)	15.93 ± 8.33	15.54 ± 8.12	-0.047
Anxiety (DASS-21 score)	11.58 ± 6.10	11.66 ± 5.70	0.014
Stress (DASS-21 score)	19.39 ± 10.18	19.13 ± 9.75	-0.025
Physical activity experience (years)	3.25 ± 1.04	3.44 ± 1.68	0.128
Training weekly load (h/week)	5.37 ± 3.41	6.36 ± 4.17	0.259
Injury occurrence (proportion)	0.425	0.596	0.348

Note: SMD values closer to 0 indicate better balance between cohorts (direction indicates which cohort has higher mean/proportion).
