# Supplementary material for: Associations Between Emotional Distress and Injury Occurrence in Physically Active Students
Source: J Clin Med. 2026 Feb 27;15(5):1822. doi: 10.3390/jcm15051822 (PMC12986016; doi:10.3390/jcm15051822)
Supplement: Supplementary file 1 [file jcm-15-01822-s001.zip › Table S2_Pearson correlation matrix for depression, anxiety, and stress.pdf]

Table S2. Pearson correlation matrix for depression, anxiety, and stress (DASS-21 subscales).

Overall sample (N=418)

	Depression	Anxiety	Stress
Depression	1.000	0.010	-0.136
Anxiety	0.010	1.000	0.018
Stress	-0.136	0.018	1.000

Males (N = 199)

	Depression	Anxiety	Stress
Depression	1.000	0.052	-0.178
Anxiety	0.052	1.000	0.009
Stress	-0.178	0.009	1.000

Females (N = 219)

	Depression	Anxiety	Stress
Depression	1.000	-0.028	-0.090
Anxiety	-0.028	1.000	0.017
Stress	-0.090	0.017	1.000
