# Supplementary material for: Associations Between Emotional Distress and Injury Occurrence in Physically Active Students
Source: J Clin Med. 2026 Feb 27;15(5):1822. doi: 10.3390/jcm15051822 (PMC12986016; doi:10.3390/jcm15051822)
Supplement: Supplementary file 1 [file jcm-15-01822-s001.zip › Table S3_Multicollinearity diagnostics.pdf]

Table S3. Multicollinearity diagnostics (VIF and tolerance) for predictors used in sex-stratified multivariable models.

Males (N = 199)

Predictor	VIF	Tolerance
Depression (DPR)	1.062	0.942
Anxiety (ANX)	1.003	0.997
Stress (STR)	1.054	0.949
Experience (EXP)	1.017	0.984
Training weekly load (TWL)	1.005	0.995

Females (N = 219)

Predictor	VIF	Tolerance
Depression (DPR)	1.008	0.992
Anxiety (ANX)	1.011	0.989
Stress (STR)	1.022	0.978
Experience (EXP)	1.029	0.972
Training weekly load (TWL)	1.048	0.954
