# Supplementary material for: Associations Between Emotional Distress and Injury Occurrence in Physically Active Students
Source: J Clin Med. 2026 Feb 27;15(5):1822. doi: 10.3390/jcm15051822 (PMC12986016; doi:10.3390/jcm15051822)
Supplement: Supplementary file 1 [file jcm-15-01822-s001.zip › Table S4_Model diagnostic.pdf]

Table S4. Model diagnostics for sex-stratified multivariable logistic regression models predicting injury occurrence (adjusted for DPR, ANX, STR, EXP, and TWL).

**Sex**	**Nagelkerke R^2**	**AUC**	**Hosmer–Lemeshow p**	**Max VIF**
Female	0.033	0.585	0.996	1.048
Male	0.062	0.615	0.505	1.062
